# Hydration Status in Older Adults: Current Knowledge and Future Challenges

**DOI:** 10.3390/nu15112609

**Published:** 2023-06-02

**Authors:** Shizhen Li, Xun Xiao, Xiangyu Zhang

**Affiliations:** Department of Geriatrics, The Second Xiangya Hospital, Central South University, Changsha 410011, China; 208202066@csu.edu.cn (S.L.);

**Keywords:** hydration status, older adults, water homeostasis, health outcomes

## Abstract

Adequate hydration is essential for the maintenance of health and physiological functions in humans. However, many older adults do not maintain adequate hydration, which is under-recognized and poorly managed. Older adults are more vulnerable to dehydration, especially those living with multiple chronic diseases. Dehydration is associated with adverse health outcomes in older adults, and acts as an independent factor of the hospital length of stay, readmission, intensive care, in-hospital mortality, and poor prognosis. Dehydration is a prevalent health problem in older adults, accounting for substantial economic and social burden. This review attempts to provide current knowledge of hydration including patterns of body water turnover, the complex mechanisms behind water homeostasis, the effects of dehydration on the health of the body, and practical guidance for low-intake dehydration in older adults.

## 1. Introduction

Water is the most essential nutrient for all living organisms, making up approximately 60% of adult body [1]. It plays a crucial role in thermoregulation, blood pressure maintenance, biochemical reaction, and transportation of nutrients into and removal of waste from cells [2]. Body water content varies slowly throughout life, being highest in infants and children, and declines with age [2]. Older adults are susceptible to dehydration, referring to a shortage of water in the body due to inadequate water intake or excessive water loss [3]. Water loss often occurs in the cases of excessive blood loss, vomiting, and diarrhea, which affects the function of many systems and can lead to acute events if it is not timely supplemented. This type of body water deficits is often called ‘salt-loss dehydration’ and is associated with diminished electrolytes in addition to reduced body water. It is distinguished from the other type of dehydration, called ‘water-loss dehydration’, where electrolyte levels are stable and relatively elevated [4]. Because inadequate water intake represents the primary cause of water-loss dehydration, European guidelines now prefer to use the term low-intake dehydration rather than water-loss dehydration [5]. Notably, low-intake dehydration is a common and chronic health condition in apparently healthy older adults, especially those that need long-term care and hospitalization [6,7]. 

Risk of dehydration increases in older people, and with increasing numbers of older people internationally, more people are at risk of developing this largely reversible condition. An observational study reported that the prevalence of dehydration ranged from 1% to 60% in community-dwelling older adults depending on different measures [8]. Amongst long-term care residents, the prevalence of dehydration was variable with rates of 28–30.5% for impending dehydration and of 20–38.3% for existing dehydration [6,9,10]. A small longitudinal study conducted on nursing home residents found that dehydration occurred in 31% of participants during a 6-month period [11]. In a prospective cohort study of 200 older adults, 37% of the participants were diagnosed as dehydrated at admission to hospital and nearly two-thirds of those remained dehydrated at 48 h after admission [12]. However, these studies were conducted based on different measures and diagnostic criteria of dehydration, which could apparently result in methodological bias. Thus, objective hydration biomarkers or suitable hydration assessment tools are critical to correctly reflect hydration status in older people in the future research. Indeed, the population selection also biased results due to the nature of heterogeneity among older people. The real incidence may be considered higher due to complex clinical conditions. Dehydration also imposes substantial economic and social burden to individuals and communities. Healthcare costs reached up to USD 1.36 billion for hospitalized older patients due to dehydration in 1996 [13]. One possible reason is that dehydration can add its own complications to multiple chronic conditions and increase medical costs indirectly.

Dehydration in older adults is associated with adverse health outcomes [14]. High-quality cohort studies have found that older adults with raised serum osmolality have an increased risk of mortality, even adjusting the key confounding factors [3]. Dehydration has been shown to affect cognitive performance, and increase risk of metabolic and renal diseases. It represents an independent factor of hospital length of stay, readmission, intensive care, in-hospital mortality, and poor prognosis [12,15,16,17,18]. 

This review attempts to provide current knowledge about hydration including patterns of water turnover, the complex mechanisms behind water homeostasis, the effects of dehydration on the health of the body, and practical guidance for low-intake dehydration in older adults. Nevertheless, there are major gaps in knowledge related to optimal water requirements and optimal hydration in older adults, and the effects of long-term systematic interventions on health outcomes.

## 2. Body Water Regulation

**Figure 1:**
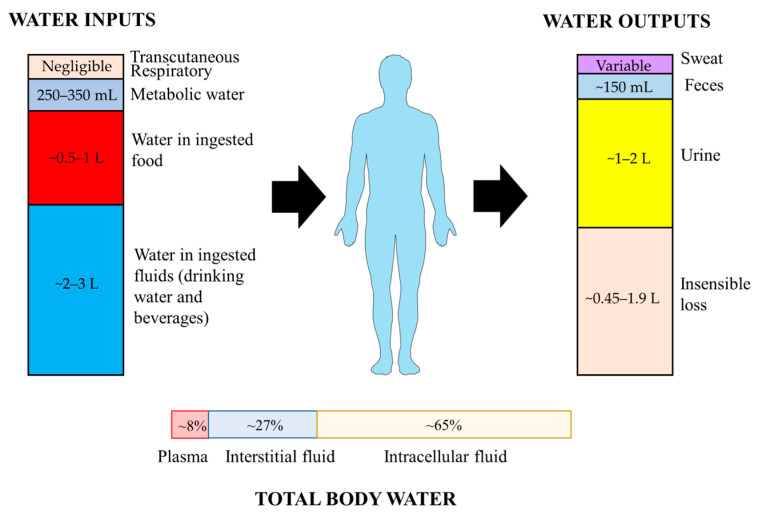
Sources of water inputs and outputs on human body and distribution of total body water.

Under normal conditions, total body water (TBW) is maintained in a dynamic equilibrium, which is regulated within 0.2% of body weight over a 24 h period [19]. TBW is distributed into two compartments, intracellular fluid (ICF) and extracellular fluid (ECF), which contain about 65% and 35% of TBW, respectively (Figure 1). The ECF compartment is further divided into blood plasma that holds blood cells in suspension, interstitial fluid that surrounds tissue cells, and transcellular fluid in lymphatic vessels and ventricles and subarachnoid space [20]. Water content varies across individuals with numerous factors including age, gender, body composition and various medical conditions [21]. For example, water typically comprises about 75% of an infant’s body weight, as opposed to 50–60% in older persons [22]. Individuals with more muscle or less fat may have higher relative TBW, as, by content, fat is only ~11% water and muscle is ~75% water [23]. 

### 2.1. Water Inputs and Outputs

It has been estimated that 5–10% of TBW, or approximately 2–2.5 L, is turned over daily. Total water inputs consist of three major parts, including drinking water, water in beverages and in food [24]. In addition, water can be also endogenously produced by the oxidation of macronutrients, and a small amount is absorbed through transcutaneous and respiratory ways. Metabolic water represents only about 250–350 mL/d in sedentary people [24]. More than two-thirds of water inputs are derived from the intake of fluids and food to offset water loss [24]. Additional water intake may be required due to high temperature, physical activity, or other conditions. Daily water intake is crucial to counterbalance with the outputs to maintain appropriate hydration status. The European Food Safety Authority (EFSA) recommended an adequate water intake of 2.0 L/d for women and 2.5 L/d for men to maintain desirable urinary osmolarity and water consumption, derived from a combined observation of drinking water, beverages, and food [25]. Assuming 20% of total water intake comes from food, women would require 1.6 L/d of daily fluid intake, and men 2.0 L/d. Accordingly, the European Society for Clinical Nutrition and Metabolism (ESPEN) also estimated daily fluid intake as 80% of total water intake for recommendation, as well as institutions below [25,26,27,28]. Recommendations for daily fluid intake by other international institutions are displayed in Table 1.

The majority of water outputs occur obligatorily via the respiratory tract, skin, and kidneys, while the rest are in the form of sweat, water in feces, and a negligible proportion of tear and sputum [21]. Daily urine volume generally approximates 1–2 L, and can be increased by consuming large amounts of fluid. The large capacity of urine output represents the primary avenue to regulate net body water balance across a broad range of fluid intake volumes [29]. Insensible water loss via respiratory tract and skin can reach ~0.45–1.9 L/d. Sweat loss varies widely and depends upon the physical activity level and environmental conditions. Gastrointestinal tract is crucial in both intake and excretion of water. The water from food (~0.5–1 L/d) and fluids (~2–3 L/d) is predominantly absorbed into blood across the highly polarized epithelial cell layer of the small intestinal mucosa [2,30]. Beside the ingested water, large amounts of digestive secretions (~8 L/d) are absorbed in the small intestine, where absorption capacity can reach up to 15 L/d [30]. Nearly 1–1.5 L water in digesta enters the colon for subsequent absorption, while only about 150 mL water is excreted in feces [31].

Notably, the majority of objective parameters on water turnover are derived from healthy adults under a stable and nearly ideal environment. Few studies have focused on water turnover in older people, especially those with multiple chronic diseases. In a small sample-size comparative study, water turnover rate was found to be faster in physically dependent older people with median water turnover of 2.2 L/d in summer and 2.1 L/d in winter, compared with an independent group (1.5 L/d and 1.6 L/d, respectively) during the same periods, which suggested an impact of physical activity on water turnover in older people [32]. Water turnover is also influenced by body composition, energy consumption, temperature, and climate conditions [21]. A recent article published in *Science* provided an in-depth understanding of body water turnover in all ages, as well as under a broad range of environments and living conditions [21]. As expected, water turnover decreased with age, and was lower in men (>40 years) and women (>65 years), respectively [21]. In this large-sample study from a global database, an equation was obtained to predict water turnover with power to explain 47.1% of variations [21]. According to the predictive equation, water turnover had a curvilinear relationship with age, reaching a peak between 20 and 40 years of age and descending after 50 years of age. It was convenient to calculate the amounts of water turnover in different ages with other available variables. For instance, individuals aged 80 had ~700 mL less water turnover than those at age 30 when holding the other variables constant. The equation is presented below: 

Water turnover (mL/d) = 1076 × Physical activity level + 14.34 × Bodyweight (kg) + 374.9 × Sex + 5.823 × Humidity (%) + 1070 × Athlete status + 104.6 × Human development index + 0.4726 × Altitude (m) − 0.3529 × Age^2^ + 24.78 × Age (years) + 1.865 × Temperature^2^ − 19.66 × Temperature (°C) − 713.1 (Physical activity level: the ratio of total energy expenditure to basal energy expenditure; Sex: 0 for women and 1 for men; Athlete status: 0 for non-athlete and 1 for athlete; Human development index: 0, 1 and 2 for high, middle and low human development index countries, respectively).

### 2.2. Physiological Mechanisms of Water Balance

Body water balance is achieved via two major mechanisms regarding the hormone arginine vasopressin (AVP) and thirst [33]. Integrated mechanisms of vasopressin and thirst collaborate to maintain osmotic homeostasis. When inadequate water intake or excessive water loss, the osmolality of ECF increases, which sends a message that ‘I AM THIRSTY’ to the brain. This signal transduction relies on specific structures in the circumventricular organs, including the subfornical organ and organum vasculosum of the lamina terminalis. Because these structures are located outside the blood–brain barrier, certain specialized neurons in the regions, called osmoreceptors can monitor changes in plasma osmolality directly and feedback appropriate activation for the generation of thirst [34]. These receptors simultaneously regulate the AVP secretion from the posterior pituitary and the osmoregulation mechanism of AVP is to be discussed in the following paragraph [35]. Voluntary drinking behavior is then driven by the sensation of thirst to replenish water [36]. Therefore, thirst can be deemed as a defense mechanism to counteract deficits of body water. However, the thirst sensation often diminishes in older adults. Thirst and responses to osmotic stimulation has been evaluated in old adults via administration of hypertonic saline or fluid restriction. Phillips and colleagues demonstrated that healthy older men deprived of water for 24 h reported no significant increase in subjective sensations of thirst or mouth dryness at post-deprivation in comparison with young controls [37]. Moreover, older men drank less water and had raised plasma osmolality compared to pre-deprivation levels. However, osmotic loading thirst was intact in healthy older people after 2 h of hypertonic saline infusion [38,39]. One reasonable explanation is that older people appear to have relatively poor responses to the slowly elevated plasma osmolality for thirst. This blunted thirst sensation is an important contributory factor to increased risk of dehydration in older people.

**Figure 2:**
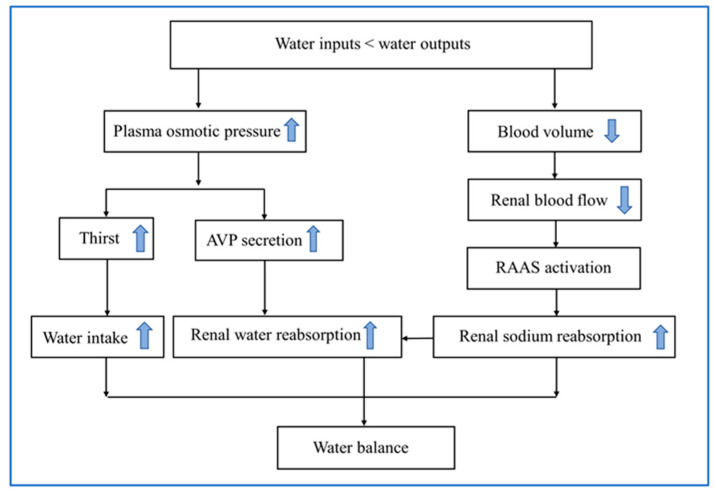
Physiological mechanisms of water balance in response to dehydration. AVP, arginine vasopressin; RAAS, renin–angiotensin–aldosterone system.

AVP, also called antidiuretic hormone, is a nine-peptide hormone. It is mainly synthesized by magnocellular neurons in the paraventricular and supraoptic nuclei of the hypothalamus, and transported within the axons into the posterior pituitary and stored there [33]. The secretion of AVP is fine-tuned via a set point of plasma osmolality within a normal range of 275–295 mOsm/kg. As plasma osmolality increases by 1–2%, AVP is released into blood stream and plasma concentration is elevated at 1 pg/mL, which leads to a remarkable decreased excretion of free water and reduction in urine volume [40]. The target of antidiuretic action of AVP is the vasopressin receptor subtype V2 receptor, which is located at the basolateral membrane of renal collecting duct cells [40]. By binding to its specific V2 receptor, AVP activates cyclic adenosine monophosphate/protein kinase A pathway, and increases the expression of the water channel protein aquaporin-2 in apical membrane of renal collecting duct cells, thus increasing water permeability in the collecting duct to resorb water [40]. This compensatory mechanism to increase water reabsorption is prior to thirst. In addition, there are other neurohumoral mechanisms responsible for maintaining water homeostasis, including the renin–angiotensin–aldosterone system [41] (Figure 2).

### 2.3. Age-Related Changes in Water Balance Regulation 

Aging is a multifactorial process characterized by progressive functional decline that affects every organ, including the kidney. The aged kidney is changed anatomically with less mass, interstitial fibrosis, and progressive nephrosclerosis, and compromised in the compensative and homeostatic regulatory function [42,43]. Evidence based on longitudinal population studies indicates that many aspects of renal function, such as creatinine clearance, glomerular filtration rate, and maximal urinary concentrating ability, decrease with advancing age. Glomerular filtration rate declines after age 40 years, and accelerates after age 65 years. The glomerular filtration rate declines approximately by half from the ages of 30 to 80 years [44]. Compared with younger adults, older people aged 60 to 79 years have an approximately 20% reduction in maximum urinary concentrating ability [45]. By age 80, there is a reduction of more than half in maximum urinary concentrating ability comparing to the youthful peak of 1100 to 1200 mOsm/kg. These changes can aggravate water deficits and dehydration in older people. 

As described above, renal ability to maintain fluid homeostasis is regulated by AVP signaling. Normal AVP secretion is essential for water reservation. However, there are contradictory results with respect to basal AVP levels in older people. Some studies reported that AVP is reduced or unchanged with advancing age [46,47,48]. In contrast, observational studies found that AVP secretion is augmented in healthy older people compare with young subjects [49]. Possible explanations for the reduction in urinary concentrating ability in older people may be the abnormal renal response to AVP, as baseline plasma AVP were strongly correlated with serum osmolality in youngers but not in older people. Interestingly, hypertonic saline infusion could raise plasma osmolality, while AVP levels in plasma rose more rapidly [39]. Furthermore, increased basal plasma AVP levels in older people are not related to age-related changes in AVP pharmacokinetics, and may reflect age-related changes in central control systems for AVP release.

## 3. Definition and Causes of Dehydration

Although there is no internationally accepted definition, dehydration is usually described as a deficit in TBW. This can be further categorized as hypotonic, isotonic, or hypertonic dehydration based on electrolyte levels. Hypotonic or salt-loss dehydration occurs when, proportionally, more salt is lost than water [2]. Isotonic dehydration occurs when both salt and water are lost equivalently from the body, for example, diarrhea. Hypertonic or water-loss dehydration can be caused by inadequate fluid intake, excessive sweating or transcutaneous evaporation, and/or vomiting [2]. A deficit of >2% body mass loss (~3% of TBW) reflects dehydration status or impending dehydration [4,50]. Daily variation in body mass of ≤1% body mass is regarded as normal, while daily fluctuations in body mass between 1% and 2% cannot be reliably related to perturbations in TBW. There is also no international consensus on the different gradations of dehydration severity [50].

Older people are more susceptible to dehydration than younger people. There are multiple contributors to dehydration among the elderly. Age-related physiological factors contributing to dehydration include blunted thirst sensation and reduced urinary concentrating ability of the kidney in older people [51]. Besides physiological changes, cognitive impairment is one of the most common psychological factors that increases the risk of dehydration with age [52]. Many older adults often forget to drink sufficient water due to memory problems caused by normal aging or diseased state, such as dementia and delirium. Impaired physical abilities, reduced mobility and inability to eat and drink also increase elderly people’s risk of dehydration, especially those living at home and in institutions [53]. Furthermore, older patients with poorly controlled diabetes are more likely to be dehydrated because of the diuretic efficacy of hyperglycosuria [52]. Those suffering from urinary incontinence may also increase vulnerability to dehydration due to self-restriction of fluid intake. Older patients with dysphagia are prone to dehydration due to poor oral intake to meet the requirements [54]. Finally, environmental factors should not to be neglected since older people have a poor capacity to adapt ambient environment [52].

## 4. Measurement of Hydration Status

Hydration status assessment in the elderly is considered complex. A Cochrane systematic review documents that some clinical symptoms and signs should not be used to indicate dehydration in older adults due to insufficient sensitivity and reliability [55]. These symptoms and signs include heart rate, skin turgor, mouth dryness, and thirst sensation. Although urine indices, such as urine gravity and urine color, are the usual extended methods to assess hydration status, there is substantial evidence regarding the ineffectiveness of urine gravity and urine color for indicating dehydration. The ESPEN practical guideline explicitly recommended direct measurement of serum or plasma osmolality, which is defined as the sum of concentrations of osmotic components, as diagnostic criteria to identify low-intake dehydration in older people [28]. The suggested threshold of serum or plasma osmolality for dehydration is 300 mOsm/kg. The direct measurement of serum osmolality is performed using freezing point depression or vapor pressure depression osmometers, but it is circumscribed in clinical practice. Alternatively, through measuring molar concentrations of dissolved molecules, a theoretical value to approximate osmolality can be obtained. The equation that osmolarity = 1.86 × (Na^+^ + K^+^) + 1.15 × glucose + urea + 14 (converted in mmol/L) is recommended to independently predict serum osmolality in older adults [56]. A cut point of 295 mOsm/L can identify most adults with low-intake dehydration with 85% sensitivity and 59% specificity [56]. There is no gold standard to assess hydration status. Although plasma osmolality with TBW assessment is regarded as the most precise and common measure to indicate hydration status, it is not practical in all settings because it requires blood samples and expensive instruments.

Bioimpedance is a portable, efficient, and non-invasive method to predict body water volumes (TBW and ECF) and body composition. It measures the impedance of entire body and its two components, namely resistance and reactance, via an electrical current that flows though water and electrolytes in the body [57,58]. This method is easy to use and inexpensive, and can provide real-time measurement of body components. However, many factors, such as the site placement of electrodes, postures, and diseased states, reduce the reliability and accuracy of this technique [57]. Moreover, the bioimpedance method remains inappropriate for measuring small changes in TBW. It appears to be inaccurate under conditions of acute change in intracellular or extracellular water [59]. Although the bioimpedance method has been utilized as an objective indicator to assess hydration status in clinical research [4,28], it is currently not useful in older people, and requires further evaluation. In addition, a wide array of engineering approaches to assess hydration status are proposed and discussed in detail by others [60].

Tracer dilution techniques are regarded as the gold standard to measure TBW using the commonly employed tracers such as deuterium oxide (D_2_O) and oxygen-18 (H_2_^18^O) [61]. By determining the tracer concentrations and elimination rate in body, TBW and water turnover can be calculated [61]. Although the tracer method is objective, accurate and reliable, it is expensive and laborious, which limits its clinical use.

Because no single indicator is entirely reliable, combined methods may have an additional efficacy for hydration status assessment. Whilst evidence is limited, clinical signs, urine indices and symptoms can also be an indicator for the risk or early stages of dehydration to augment diagnostic accuracy. Furthermore, novel methods or technologies with more availability, accuracy and convenience need to be developed in the future.

## 5. Water Intake, Hydration and Health Outcomes

**Figure 3:**
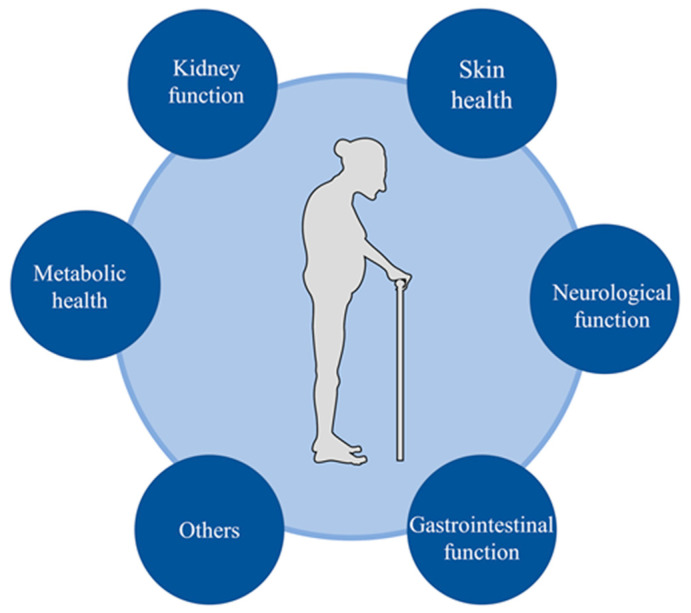
Hydration and major health outcomes in older people.

It is generally assumed that proper hydration to maintain serum osmolality within reference ranges is essential for homeostasis and life. In experimental animals, suboptimal hydration could contribute to metabolic impairment, promote age-related degenerative changes, and shorten lifespan [62]. In humans, chronic lifelong mild dehydration was a potential determinant for the development of the age-related degenerative diseases by analyzing data from the “Atherosclerosis Risk in Communities” study [62,63,64]. Chronic dehydration is associated with an increased risk of degenerative diseases or diminished physiological function, and improved hydration status may positively affect various chronic diseases (Figure 3). 

### 5.1. Skin Health

Human skin is the first line of barrier to protect our bodies from external challenges (e.g., physical, chemical, and pathogenic insults) [65]. It is of importance in the maintenance of body temperature and regulation of water balance. Water is essential for the normal skin function [66]. Inadequate hydration of the skin and especially its outer layer, the stratum corneum, will impair barrier function, normal desquamation, and physical characteristics [67]. The skin undergoes progressive degenerative change with advancing age. A widely accepted interpretation of dry skin is a lack of water in the stratum corneum [68]. A recent study demonstrated that reduced stratum corneum hydration in older people might contribute to inflammaging with certain elevated serum cytokine levels [69]. In a recent pilot clinical trial, improved hydration of the stratum corneum with topical emollient could impede the progression of cognitive impairment in older people [70]. Research between skin hydration and health is sparse, while a number of studies are focused on interventions to improve skin hydration. In a systematic review, stratum corneum hydration increased following additional water intake of 2 L/d over a period of 30 days or 1 L/d for a period of 42 days [71,72,73]. Although quantity and methodological quality exist in these studies, this common approach to improve skin hydration by drinking more water is well-accepted. In short, skin is of vital importance for health and well-being, but it remains largely unknown how skin hydration affects health.

### 5.2. Neurological Function

Cognitive impairment is an important global health problem of special concern in old people. It has been reported that mild dehydration can impair cognitive function, leading to disruptions of concentration, alertness and short-term memory [74]. Moreover, dehydration can also cause initial symptoms of headaches, fatigue and lethargy, and diminish one’s mood among middle-aged people [14,75,76,77]. Several studies have demonstrated that cognitive performance is associated with hydration status in older people [78,79]. In a small sample study of community-dwelling older people, Suhr and colleagues found that lower hydration status was related to slow psychomotor processing speed and poorer performance on attention and memory, even after adjusting for age, education and systolic blood pressure [80]. In another study conducted by Suhr and colleagues, TBW was related to working memory performance on community-dwelling older women [81]. However, the effects of dehydration on cognition are inconsistent in some cases. Białecka-Dębek and colleagues demonstrated that there was no significant relationship between cognitive performance and good hydration status measured via morning urine specific gravity in the seemingly healthy older people [78]. In the record from the 2011–2014 cycles of Nutrition and Health Examination Survey (NHANES), hydration status and water intake were moderately associated with attention and processing speed among older females, but not observed among older men [82]. As described in previous reviews, many studies on hydration and cognitive function were heterogeneous in measurements, methodology and outcomes. Furthermore, these studies were observational and short-term with a relatively small sample size, which could not investigate the causal relationship between hydration and cognitive function. On the other hand, older people with cognitive impairment, including dementia, may experience difficulty with self-care, which increases the risk of dehydration. To date, only one multi-year prospective cohort study was conducted to assess the association of water intake and hydration status with cognitive performance. The study revealed that poorer hydration status was associated with a greater decline in global cognitive function over a 2-year period in 1957 older Spanish adults [83]. Nonetheless, few studies have examined the relationship between water recharge and cognitive function. It is of valuable importance to investigate whether an evaluation of water consumption or good hydration status for a longer period could improve cognitive performance. 

### 5.3. Gastrointestinal Function

The normal gastrointestinal function is essential to maintain the wellness of human body. Gastrointestinal function undergoes modest changes with aging. However, evidence linking gastrointestinal function and hydration status in older individuals is limited. Constipation, as one of the most frequent gastrointestinal disorders, is often encountered in clinical practice among older people, which is characterized by slow intestinal transit, difficult defecation, and abnormal fecal retention [84]. Inadequate water intake is regarded as a common cause of constipation, and increasing water intake (1.5–2 L/d) is the first line treatment in the opinion of most health professionals, even though this is not based on solid scientific arguments. It seems likely that water has a laxative action, especially that which is rich in magnesium and sulfates [85]. In 796 participants of the New Mexico Elderly Health Survey, a trend toward a higher constipation frequency was associated with a low fluid intake [86]. In an observational study containing 21,012 older people, 7% of them developed constipation, and low fluid consumption was the second predictor of constipation [87]. There are no randomized controlled trials to evaluate the effect of water supplementation alone for the treatment of constipation, although a total of 1.5–2 L/d of water supplementation improved stool frequency in middle-aged adults with a high-fiber diet [85,88]. Low water intake is an important risk factor of functional constipation in older people [89]. Participants in the middle upper quartile for water intake had a decreased likelihood of constipation when compared with those in the lowest quartile [90]. Numerous studies recommend increasing fluid intake to treat constipation. Few studies have reported that increasing liquid volume is effective as a treatment in well-hydrated subjects with chronic constipation. Therefore, more evidence is required to determine whether there are benefits or adverse effects from this recommended high fluid intake. 

The gut microbiota is an integral part of the human body, performing some basic functions in the immunological, metabolic, cardiovascular, and gastrointestinal regulation [91]. Hydration biomarkers were related to intestinal mucus homeostasis in adults. In addition, it has been reported that the gut microbiota changed to resist the fluid change related to temperature change and exercise, suggesting the subtle effects of body hydration conditions on the microbiota [92,93,94]. Gut microbiota has become an area of increasing interest during the last decade, and the interplay between gastrointestinal function and hydration deserves in-depth research.

### 5.4. Kidney Function

The kidney is the central organ involved in regulating water balance and removing waste from the body [95]. Large amounts of ultrafiltrate of plasma are filtered across the glomerular barrier. Most of the water and other small molecular substances are reabsorbed and concentrated urine is formed [95]. However, the renal ability to concentrate urine declines with age, which is one of the contributors to geriatric dehydration. Therefore, a question has been posed reversely regarding whether water intake or hydration can have an impact on kidney function in older people. Several observational studies have found that increased water intake was associated with better kidney function in patients with chronic kidney disease (CKD) and those at risk of CKD [96]. In a randomized clinical trial of 631 CKD patients with a mean age for 65 years, increased water intake (compared with usual fluid intake alone) did not slow a decline in kidney function measured via eGFR over 1 year [97]. In the CKD-REIN cohort study, the relation between plain water intake and progression to kidney failure appears to be U-shaped in patients with CKD [98]. Both low and high intake may not be beneficial in CKD. To assess the effect of increased daily water intake on healthy people, Yumi Nakamura and co-authors conducted an open-label, two-arm, randomized controlled trial on 174 middle-aged and aged subjects, water supplementation reduced blood urea nitrogen concentration, and suppressed estimated glomerular filtration rate reduction [99]. More clinical trials are needed to determine if supplemental water can safely slow the deterioration of kidney function in CKD patients. With regard to dialysis patients, it is apparent that water intake should be restricted to avoid fluid accumulation. Overall, there is a lack of evidence to claim positive impacts of population fluid intake recommendations on aged kidney health.

### 5.5. Metabolic Health

Lifestyle interventions have primarily been recognized as a fundamental strategy to prevent metabolic diseases, especially diabetes mellitus [100,101]. Recent studies demonstrated that water intake or hydration status play a unique role in metabolic diseases. Water as a substitute for sugar- and calorie-containing beverages has a potential to reduce the risk of type 2 diabetes. Notably, the nature of hydration may contribute to metabolic health independently. Epidemiological evidence has demonstrated that low daily water intake was associated with increased diagnosis of hyperglycemia [102]. In a 9-year follow-up study, water intake was inversely and independently associated with new-onset risk of hyperglycemia [103]. In observational and interventional studies, it has been implicated that the hydration–AVP axis is involved in the pathophysiology of metabolic diseases. who habitually drink fewer fluids tend to have higher levels of AVP in bloodstream. Individuals with higher AVP are at higher new-onset risk of type 2 diabetes and components of the metabolic syndrome after adjusting for known risk factors [104]. Individuals who habitually drink less water tend to have higher levels of copeptin, a surrogate marker of AVP. After receiving 1.5 L/day of water supplementation for 6 weeks, the high copeptin levels and glucose concentration decreased [105]. Therefore, it is suggested that AVP corelated to a dehydrated state may play a crucial role in the development of metabolic diseases. Improving hydration by increasing water intake is a simple and inexpensive potential strategy to prevent the development of metabolic dysfunction, and further studies with large-scale, longer-period conditions are needed.

## 6. Management and Treatment of Low-Intake Dehydration

The new 2022 ESPEN practical guideline on clinical nutrition and hydration in geriatrics provides evidence-based recommendations and consensus on the treatment and prevention of geriatric dehydration [28]. Since low-intake dehydration is common in older people, all older persons are encouraged to consume adequate amounts of drinks, not merely water [28]. The recommended adequate water intake from drinks is 1.6 L/d for female and 2 L/d for males, according to a calculation of 80% of daily total fluid requirement recommended in EFSA. However, individual fluid needs are highly variable, which take into account exercise, environment and comorbidities. Additional water intake may be needed in higher temperatures, during greater physical activity [106,107], or due to excessive fluid losses (e.g., fever, diarrhea, and vomiting) [108]. In contrast, in the settings of heart failure, renal dysfunction, or severe liver diseases, geriatric patients with fluid retention should restrict fluid intake.

As for the choice of drinks, the ESPEN guideline recommends that older people can consume appropriate drinks with a hydrating effect according to their preferences, such as tea, coffee, fruit juices or alcohol drinks [28]. Solid evidence supports that neither coffee nor alcoholic drinks of up to 4% alcohol have a dehydrating effect. There concerns regarding whether preferred drinks are suitable to replenish water in older people with special medical conditions. For example, sugar intake needs to be restricted for diabetes patients, and the consumption of sugar-sweetened beverages was positively associated with higher risk of incident diabetes [109]. Importantly, long-term effects of these beverages on health outcomes other than hydrating action should be verified via longitudinal studies. 

Multicomponent strategies for older people living in residential care may be effective and have been recommended in the ESPEN guideline [28,110]. These strategies should include high availability, varied choice, and frequent offering of drinks. Increased staff awareness, assistance with drinking, and support using the toilet may be effective. In addition, strategies to support adequate hydration should be developed with the involvement of older residents, staff, management, and policymakers [28].

For the treatment of low-intake dehydration, older people are encouraged to drink more fluids as they prefer. If they appear unwell, older adults can receive subcutaneous or intravenous fluid supplement in parallel with encouraging oral fluid intake. When older persons are severely dehydrated and require aggressive hydration, the administration of intravenous fluid is an adequate choice to quickly replenish body water [28]. During the rehydration process, comprehensive assessment including hydration status should be carried out regularly for an individualized drinking strategy.

## 7. Challenges and Future Directions

Dehydration is generally regarded as the state of body water deficit. Acute changes of body weight have been used as the gold standard to diagnose dehydration. However, it is unrealistic to carry out the diagnostic criteria in older people. Many older persons develop a hypohydrated state over the course of a long time, and body weight changes slowly throughout the life therefore, baseline euhydrated body weight is rarely available to accurately ascertain the degree of water deficits. Although the serum osmolality or osmolarity is an objective indicator recommended to diagnose geriatric low-intake dehydration in older persons, several questions remain unanswered. What is a real well-hydrated state? How much water should we drink to reach such a real well-hydrated state? Shall we drink more water even if habitual water intake meets the guideline recommendation? Comparing the 10th (1694 mL/d) and the 90th (7934 mL/d) percentile of water intake distribution in the US, these have a nearly detected plasma osmolality of 279 and 280 mmol/kg, respectively [111]. Individuals who habitually drink either low or high amounts of water have similar plasma osmolality. This means plasma osmolality is maintained within a narrow range, despite large variations in normal water intake and loss. 

Low-water intake intuitively leads adverse actions on health outcomes as we believe a theoretical point of view that states: ‘Drink at least eight glasses of water a day’ [112]. In reality, a significant number of studies are in favor of the abovementioned opinion but lack solid evidence, especially for long periods of study. There are major gaps in the knowledge related to the effects of long-term systematic interventions on health outcomes. Large-scale studies and randomized control trials are necessary to investigate how increased water intake impacts health and well-being. In addition, the traditional standard device to measure serum osmolarity is inconvenient and relatively large, and is mainly used in hospital settings. Novel portable device that can be easily applied in community-dwelling residents need to be developed to measure hydration. General practitioners or carers should make a comprehensive evaluation to assess hydration status in older adults, rather than focusing on the value of serum osmolarity, including thirst, urine color, comorbidities and other factors that may provide a hint of impending dehydration or risk of dehydration. Rehydration therapy is particularly difficult in the context of unawareness within that population. Furthermore, a recommended volume of daily water drinking is not yet determined in older patients with certain diseases, especially those with heart failure, kidney disease, or other causes of fluid overload. It is fundamentally important to determine ‘true’ water requirements and individualized recommendations for older adults.

## Figures and Tables

**Table 1:** Dietary fluid intake reference recommended by international groups.

Institution	Year	Daily Fluid Intake	Age
EFSA [25]	2010	≥1.6 L for women ≥2.0 L for men	Adults (≥18 years)
IOM [26]	2014	2.2 L for women 3.0 L for men	Adults (≥50 years)
CNS [27]	2019	1.5 L for women 1.7 L for men	Older adults (≥65 years)
ESPEN [28]	2022	≥1.6 L for women ≥2.0 L for men	Older adults (≥65 years)

WHO, World Health Organization; EFSA, European Food Safety Authority; IOM, Institute of Medicine; CNS, Chinese Nutrition Society; ESPEN, European Society for Clinical Nutrition and Metabolism.

## Data Availability

Data sharing not applicable.
